# Diagnostic value of ultrasound for community-acquired pneumonia in children and its correlation with serum PCT level and PCIS

**DOI:** 10.1097/MD.0000000000039590

**Published:** 2024-10-25

**Authors:** Qing-Zhong Liu, Zi-Qiang Feng, Kai-Wei Huang, Zi-Jiang Yang, Li-Qin Xu, Yuan-Yuan Shen

**Affiliations:** a Department of Pediatrics, Taihe Hospital, Hubei University of Medicine, Shiyan, China; b Department of Surgery, Shiyan Maternal and Child Health Hospital, Shiyan, China; c Department of Ultrasound, Taihe Hospital, Hubei University of Medicine, Shiyan, China; d Clinical Skills Teaching and Training Center, Hubei University of Medicine, Shiyan, China.

**Keywords:** community-acquired pneumonia in children, diagnostic value, lung ultrasound, pediatric critical illness score, serum procalcitonin

## Abstract

**Objective:**

This study aimed to evaluate the diagnostic value of ultrasound for community-acquired pneumonia (CAP) in children.

**Methods:**

Clinical information of children diagnosed with CAP and a control group of healthy children was collected, and lung ultrasound detection was performed. The lung ultrasound score (LUS) was assessed, and venous blood samples were collected. Serum indexes, including white blood cell count, were analyzed using an automatic immunoassay analyzer, while serum procalcitonin (PCT) level was measured using an enzyme-linked immunosorbent assay. The pediatric critical illness score (PCIS) was also performed for all subjects.

**Results:**

White blood cell count, absolute neutrophil count, and respiratory index were significantly higher in the CAP group than those in the control group, while the oxygenation index was markedly lower. Ultrasound detection results showed that the CAP group exhibited significantly higher detection rates of pleural effusion, interstitial lung changes, lung consolidation, B-lines, air bronchogram signs, and reduced or absent lung sliding signs compared with the control group. In addition, the LUS and PCT levels were markedly higher in the CAP group, while the PCIS was notably lower. Further analysis exhibited that the LUS in the CAP group was significantly positively correlated with PCT levels and negatively correlated with PCIS. The receiver operating characteristic curve indicated that the area under the curve of LUS for diagnosing children with lung infection was 0.841.

**Conclusion:**

LUS is closely related to serum PCT level and PCIS. Lung ultrasound detection demonstrates high sensitivity and specificity, indicating its valuable clinical diagnostic utility for CAP in children.

## 1. Introduction

Community-acquired pneumonia (CAP) is a leading cause of hospitalization and mortality worldwide,^[1]^ particularly affecting those under 5 years of age.^[2,3]^ CAP has been reported to be an infectious disease caused by bacterial infections or viral invasions.^[4]^ Bacterial pathogens such as *Streptococcus pneumonia*, *Haemophilus influenza*, *Streptococcus pyogenes*, and *Staphylococcus aureus*,^[5]^ as well as respiratory viruses such as respiratory syncytial virus,^[6]^ are common causes of CAP in children. Despite the high prevalence and severe outcomes associated with CAP, diagnosing the causative agents remains challenging. In addition, the overlap of CAP symptoms with other respiratory conditions complicates accurate diagnosis based solely on clinical signs and symptoms.^[7]^ Therefore, it is necessary to improve current diagnostic strategies to address the hazards caused by CAP in children.

Traditionally diagnostic methods, including chest X-ray (CXR), have limitations in specificity and sensitivity^[8]^ and pose risks such as radiation exposure.^[9]^ Hence, there is a critical need for safer and more effective diagnostic tools. Lung ultrasound has emerged as a promising alternative, offering benefits such as the absence of ionizing radiation, cost-effectiveness, and time efficiency. Studies have suggested that lung ultrasound has higher specificity and sensitivity compared with CXR in the diagnosis of CAP in children (78.5% and 95.2%, respectively).^[10]^

This study aims to evaluate the diagnostic value of ultrasound for CAP in children, correlating ultrasound results with the serum level of procalcitonin (PCT), a promising biomarker for bacterial infections, and pediatric critical illness score (PCIS). These findings will contribute to the development of more accurate and safer diagnostic strategies for pediatric CAP.

## 2. Materials and methods

### 2.1. Study subjects

A total of 106 children diagnosed with CAP and treated at our hospital from April 2023 to October 2023 were retrospectively included in the study. In addition, 106 healthy children who received pediatric care in the same period served as the control group. This study was approved by the ethics committee of Taihe Hospital.

### 2.2. Inclusion and exclusion criteria

The inclusion criteria are given as follows: diagnosis based on clinical symptoms and signs; main clinical manifestations of fever, cough, and shortness of breath; significant rales on lung auscultation; and age between 6 months and 6 years.

The exclusion criteria included the following: unstable vital signs, pulmonary tuberculosis, severe cardiopulmonary dysfunction, and incomplete case data.

### 2.3. Collection of basic clinical information

Basic information collected from the patients included age, gender, weight, height, heart rate, and respiratory rate. The oxygenation index (OI) and the respiratory index were calculated using respiratory indices.

### 2.4. Lung ultrasound score standards for lung ultrasound detection

Lung ultrasound detection was performed using a portable color Doppler ultrasonic diagnostic device (Siemens AG, Germany, #AU800) with a probe frequency of 9 to 15 MHz. The patients were positioned supine. The chest was divided into 4 regions according to the sternal angle plane and the mid-clavicular line. These 4 regions were further divided into anterior, middle, and posterior regions, respectively, using the axillary front line and the mid-axillary line, totaling 12 regions. Ultimately, the 12 regions of the lung were scored.

The following abnormal signs were identified during lung ultrasound detection: B-lines (ring-down artifacts), formed by the reflection of ultrasound waves at the air-liquid interface of the alveoli; lung consolidation, where inflammation leads to exudation in the lung, creating ultrasound signs similar to parenchymatous organs; air bronchogram sign, characterized by the appearance of solid changes in the lung tissue with the flickering and swinging of air inside the lung tissue during breathing; pleural line abnormalities, where the pleural line exceeds 2 mm in thickness, appearing irregular, blurred, or disappeared; pleural effusion, with fluid width in the pleural cavity exceeding 3 mm; and reduced or absent lung sliding sign, where the horizontal sliding of the pleura is absent due to significant exudation from lung inflammation.

A scoring system was used: a score of 0 for lung sliding sign with A-lines or <2 isolated B-lines; 1 point for visible A-lines and scattered B-lines; 2 points for multiple and typical B-lines; 3 points for multiple and confluent B-lines; and 4 points for visible tissue imaging in the area of lung consolidation accompanied by typical air bronchograms, abnormal pleural lines, and pleural effusion. The lung ultrasound score (LUS) was recorded for all children within 48 hours of admission.^[11]^

### 2.5. Serum index detection

Except for routine examinations, the venous blood (5 mL) was taken from each hospitalized child, while peripheral vascular access was established. Subsequently, the blood samples were put into the EDTA tubes and centrifuged at 3000 r/min for 10 minutes to separate the serum. Then, the white blood cell (WBC) count, the absolute neutrophil count, and the level of hemoglobin (Hb) in the serum of each group were analyzed using an automatic immunoassay analyzer (E0BASE411, GE).

The concentration of PCT was detected using an enzyme-linked immunosorbent assay kit (Mlbio, Shanghai, China) in strict accordance with the instructions. Specifically, 50 µL of PCT standard and 50 µL of supernatant samples were put into the relevant wells and incubated for 30 minutes. Next, 50 µL of streptavidin-HRP was put into each well of the plate. After the incubation and washing, 50 µL of solution A and solution B were added and incubated for 15 minutes. After the incubation, 50 µL of stop solution was added. The absorbance was measured at 450 nm by a multifunctional enzyme labeling instrument (Ensight, PerkinElmer).

### 2.6. Pediatric critical illness score

The PCIS is a comprehensive scoring system used to assess the severity of illness in pediatric patients. It is based on several physiological and clinical parameters, including vital signs (such as body temperature, heart rate, respiratory rate, and blood pressure), laboratory results (such as WBC and Hb), neurological status, secretion characteristics, OI, CXR findings, and bacterial culture. The total score on this scale was 100 points. The lower scores were involved in the more severe symptoms and conditions of lung infection, reflecting a higher severity of illness.^[12]^

### 2.7. Data analysis

The data collected in this study were processed using SPSS 25.0 software. The enumeration data were expressed as n (%). The χ^2^ test was used for the difference between the 2 groups. The measurement data were represented as mean ± standard deviation, and the differences were determined by a *t* test between the 2 groups. The Spearman correlation coefficient was utilized to analyze the LUS and the correlation of PCIS and PCT levels. The diagnostic value of LUS was analyzed using the receiver operating characteristic (ROC) curve. *P *< .05 was considered statistically significant.

## 3. Results

### 3.1. Basic information analysis

There were 59 male children (55.66%) and 41 female children (44.34%) in the CAP group, and 65 boys (61.32%) and 41 girls (38.68%) were included in the control group. As shown in Table 1, there were no significant differences in gender, age, weight, height, heart rate, respiratory rate, and Hb level between the 2 groups. However, compared with the control group, the CAP group had significantly higher levels of WBC, absolute neutrophil count, and respiratory index in the serum, while the OI was significantly lower (*P *< .001).

**Table 1:** Comparison of basic information between the 2 groups of children.

	Control group (n = 106)	CAP group (n = 106)	χ^2^/t	*P*
Gender, %			0.699	.403
Male	65 (61.32)	59 (55.66)		
Female	41 (38.68)	47 (44.34)		
Age, yr	3.47 ± 1.34	3.72 ± 1.27	1.425	.156
Weight, kg	15.85 ± 1.60	15.87 ± 2.12	0.088	.930
Height, cm	104.38 ± 4.91	105.25 ± 4.82	1.299	.195
Heart rate, bpm	113.81 ± 6.59	113.59 ± 6.99	-0.233	.816
Respiratory rate, bpm	27.65 ± 2.13	27.85 ± 2.48	0.624	.533
Hb, g/L	129.15 ± 6.56	128.82 ± 7.45	-0.345	.730
WBC (× 10^9^/L)	6.29 ± 1.88	12.67 ± 1.90	24.586	<.001
NEUT (× 10^9^/L)	5.62 ± 2.27	9.63 ± 2.96	11.078	< .001
RI	1.44 ± 0.34	2.46 ± 0.41	19.681	<.001
OI, mm Hg	335.36 ± 11.83	239.12 ± 12.33	-57.979	<.001

Data were expressed as mean ± standard deviation or n (%).

CAP = community-acquired pneumonia, Hb = hemoglobin, NEUT = absolute neutrophil count, OI = oxygenation index, RI = respiratory index, WBC = white blood cell count.

### 3.2. Analysis of lung ultrasound detection

Lung ultrasound detection was performed on children in both the CAP and control groups, with results summarized in Table 2. In the CAP group, 18 cases of pleural effusion (16.98%), 20 cases of interstitial lung changes (18.87%), 8 cases of lung consolidation (7.55%), 43 cases of B-lines (40.57%), 39 cases of air bronchogram sign (36.79%), and 32 cases of reduced or absent lung sliding sign (30.19%) were observed. In the control group, pleural effusion was found in 5 cases (4.72%), interstitial lung changes in 9 cases (8.49%), B-lines in 19 cases (17.92%), air bronchogram sign in 17 cases (16.04%), and reduced or absent lung sliding sign in 11 cases (10.38%). Notably, lung consolidation was not found in the control group. Statistical analysis indicated that compared with the control group, the CAP group exhibited higher detection rates of pleural effusion, interstitial lung changes, lung consolidation, B-lines, air bronchogram sign, and reduced or absent lung sliding sign (*P* < .001 or *P* < .05).

**Table 2:** Comparison of ultrasound results between the 2 groups of children.

	Control group (n = 106)	CAP group (n = 106)	χ^2^	*P*
Pleural effusion, %	5 (4.72)	18 (16.98)	8.242	.004
Interstitial lung changes, %	9 (8.49)	20 (18.87)	4.834	.028
lung consolidation, %	0 (0)	8 (7.55)	6.365	.012
B-lines, %	19 (17.92)	43 (40.57)	13.130	<.001
Air bronchogram sign, %	17 (16.04)	39 (36.79)	11.745	<.001
Reduced or absent lung sliding sign, %	11 (10.38)	32 (30.19)	12.865	<.001

CAP = community-acquired pneumonia.

### 3.3. Comparison of LUS, serum PCT level, and PCIS

Furthermore, serum PCT level, LUS, and PCIS were measured. The CAP group showed significantly higher LUS (15.73 ± 4.37 vs 9.67 ± 4.07) and serum PCT levels (0.97 ± 0.40 vs 0.11 ± 0.06 ng/mL) in comparison with the control group, while the PCIS (80.73 ± 12.76 vs 94.78 ± 13.2) was markedly lower (*P *< .001; Table 3).

**Table 3:** Comparison of LUS, serum PCT level, and PCIS between the 2 groups of children.

Group	n	LUS (points)	Serum PCT, ng/mL	PCIS (points)
Control group	106	9.67 ± 4.07	0.11 ± 0.06	94.78 ± 13.2
CAP group	106	15.73 ± 4.37	0.97 ± 0.40	80.73 ± 12.76
t		10.450	21.837	−7.882
*P*		<.001	<.001	<.001

CAP = community-acquired pneumonia, LUS = lung ultrasound score, PCIS = pediatric critical illness score, PCT = procalcitonin.

### 3.4. Correlation analysis of LUS with serum PCT and PCIS

**Figure 1.**
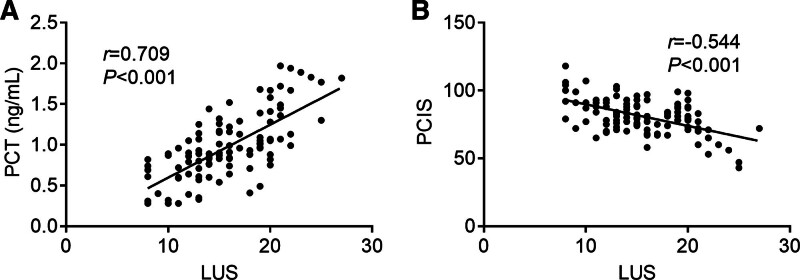
Correlation analysis of lung ultrasound score (LUS) with serum procalcitonin (PCT) level and pediatric critical illness score (PCIS). (A) Correlation analysis between LUS and serum PCT level. (B) Correlation analysis between LUS and PCIS.

The association between LUS scores and serum PCT level, as well as PCIS, was analyzed in this study to observe the correlation between ultrasound findings in CAP in children and these parameters. The results revealed a significant positive correlation between LUS and serum PCT level (Fig. 1A; *R* = 0.709; *P *< .001) and a significant negative correlation between LUS and PCIS (Fig. 1B; R = −0.544; *P *< .001).

### 3.5. Clinical value of LUS in the diagnosis of CAP

**Figure 2.**
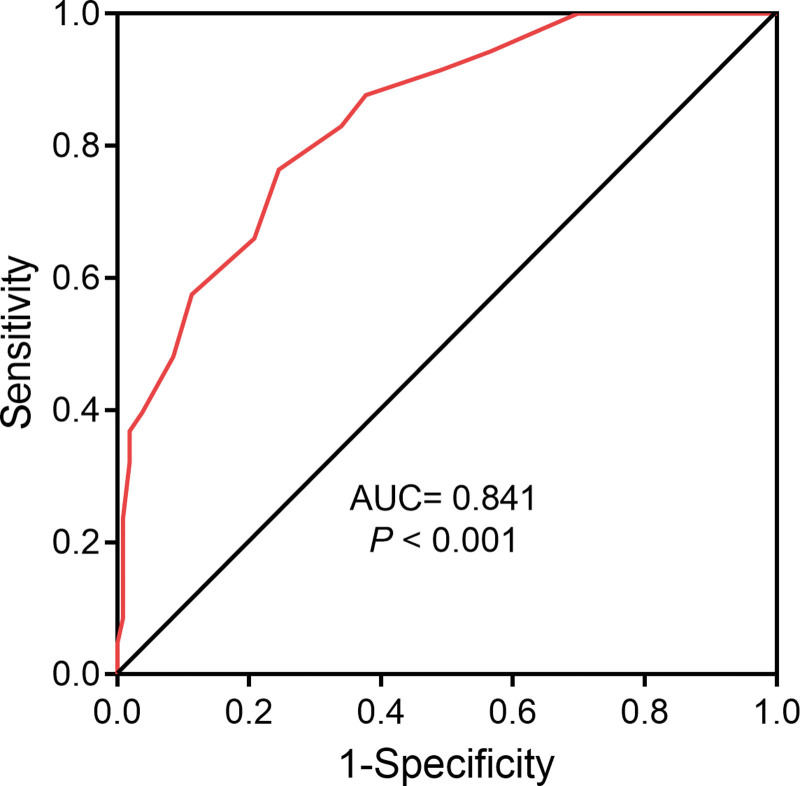
Clinical value of lung ultrasound score (LUS) in the diagnosis of community-acquired pneumonia in children. The clinical value of LUS was analyzed by the receiver operating characteristic curve.

ROC curve analysis was conducted to evaluate the diagnostic value of ultrasound in CAP in children, using clinical symptoms and signs as diagnostic criteria alongside LUS. As revealed in Figure 2, LUS demonstrated a sensitivity of 76.4% and a specificity of 75.5% for diagnosing lung infection. The area under the ROC curve was 0.841, indicating a significant diagnostic value (*P* < .001).

## 4. Discussion

CAP can be caused by bacterial or viral infections, making its clinical diagnosis challenging. According to the 2011 Pediatric Infectious Diseases Society/Infectious Diseases Society of America guidelines for CAP, it was recommended that blood cultures could be performed for hospitalized children with CAP.^[13]^ However, blood cultures yield pathogens in only 2% to 7% of children with CAP.^[14]^ Lower respiratory tract culture was usually used in clinical practice because bronchoalveolar lavage is rarely suitable for diagnosing patients with CAP. This method requires invasive procedures to obtain pleural effusion specimens, which may cause secondary harm to children.^[15]^ In addition, serotype-specific urinary antigen tests, although validated in adults, have uncertain usefulness in children.^[16]^ Thus, diagnosing bacterial pneumonia remains fraught with challenges.

Polymerase chain reaction (PCR) detection from upper respiratory tract samples is common for viral pneumonia diagnosis, while its efficacy may diminish in lower respiratory tract infections.^[17]^ Notably, the overlap of bacterial and viral infections in CAP complicates microbiological diagnosis, reducing the accuracy of these methods.^[18]^ Hence, it is necessary to test viral and bacterial pathogens in CAP simultaneously as their coexistence often correlates with severe clinical course.^[19]^

Imaging observation plays a crucial role in the diagnostic process of pneumonia, with ultrasound emerging as a promising tool due to its noninvasive nature and accuracy in detecting pneumonia-related changes.^[20]^ Studies have demonstrated ultrasound’s effectiveness in diagnosing pleural effusion in children with CAP, showing high negative predictive value (93%–94%) and positive predictive value (98.1%–100%).^[21,22]^ In addition, ultrasound could predict severe necrotizing pneumonia by correlating impaired perfusion with necrosis severity observed in computed tomography.^[23]^ While lung ultrasound is a valuable, noninvasive tool for detecting pneumonia-related changes, it has limitations in differentiating between viral and bacterial pneumonia. Ultrasound findings such as pleural effusion, interstitial changes, and lung consolidations are common to both types of pneumonia, making it challenging to distinguish between them based solely on ultrasound. This limitation underscores the need for complementary diagnostic tools, such as microbiological, serological, or PCR-based methods, to accurately identify the causative agents of pneumonia.

Notably, although microbiological, serological, and PCR-based tests are valuable for accurately identifying pneumonia pathogens, their use in routine clinical practice can be challenging. These methods require specialized laboratory facilities, trained personnel, and longer processing times, which may not be readily available in all healthcare settings. In addition, the need for high-quality samples and the risk of contamination can further complicate their routine application. Despite these challenges, integrating such diagnostic approaches could enhance the precision of pneumonia diagnosis and treatment.

In this study, children with CAP had higher rates of pleural effusion, interstitial lung changes, lung consolidation, B-lines, air bronchogram signs, and reduced or absent lung sliding signs compared with healthy controls, supporting ultrasound diagnostic role. The ultrasound results were scored, and the LUS was significantly higher in the CAP group in contrast to the control group. Our findings also highlighted the correlation between LUS, serum PCT level, and PCIS. PCT, a precursor to calcitonin, is typically produced in response to bacterial infections and has been linked to pneumonia severity.^[24]^ Studies suggest that elevated PCT level correlates with bacteremic CAP in adult pneumonia and are associated with inflammatory markers in pediatric CAP.^[25,26]^ Ratageri et al^[27]^ found the sensitivity and specificity of PCT (>0.5 ng/mL) for pneumonia were 29.7% and 87.5%, respectively, emphasizing its role in identifying pleural effusion and severity. Recent research has highlighted the potential diagnostic value of PCT and certain neuropeptides in distinguishing viral from bacterial pneumonia in children.^[28]^ This article underscores the utility of serum PCT levels, aligning with our findings that elevated PCT levels are associated with pneumonia severity. In our study, the serum PCT level in the CAP group was significantly higher as opposed to the control group, and the PCIS in the CAP group was notably reduced. PCIS, a composite score reflecting pediatric critical illness, accurately gauges pneumonia severity based on objective indicators.^[29]^ Similar to previous findings, our study indicates PICS correlates with systemic damage ratio, underscoring its utility in assessing pediatric pneumonia severity.^[30]^

In summary, while lung ultrasound and PCT have confirmed diagnostic value in children with CAP, a significant limitation of our study is the lack of identification of the causative agents of pneumonia using microbiological, serological, or PCR-based tests. This omission restricts our ability to accurately differentiate between viral and bacterial pneumonia, which is crucial for tailoring appropriate treatment strategies. Therefore, future studies should incorporate these diagnostic methods and PCT measurements to provide a more comprehensive understanding of the pneumonia etiology and improve the specificity of treatment interventions.

## 5. Conclusion

Overall, our results displayed that the LUS is an effective method to assess symptoms in children with CAP. Besides, the LUS is closely related to the serum PCT level and the PCIS in children. Lung ultrasound detection demonstrates high sensitivity and specificity, underscoring its clinical diagnostic value for CAP in children.

## Author contributions

**Conceptualization:** Qing-Zhong Liu, Yuan-Yuan Shen.

**Formal analysis:** Qing-Zhong Liu, Yuan-Yuan Shen.

**Supervision:** Qing-Zhong Liu, Yuan-Yuan Shen.

**Writing – original draft:** Qing-Zhong Liu, Yuan-Yuan Shen.

**Writing – review & editing:** Qing-Zhong Liu, Yuan-Yuan Shen.

**Data curation:** Zi-Qiang Feng, Kai-Wei Huang.

**Investigation:** Zi-Qiang Feng, Kai-Wei Huang.

**Project administration:** Zi-Qiang Feng, Kai-Wei Huang.

**Visualization:** Zi-Qiang Feng, Kai-Wei Huang.

**Methodology:** Zi-Jiang Yang, Li-Qin Xu.

**Software:** Zi-Jiang Yang, Li-Qin Xu.

**Validation:** Zi-Jiang Yang, Li-Qin Xu.
